# Switching to doravirine-based antiretroviral therapy without genotypic resistance tests in virologically suppressed people with HIV

**DOI:** 10.1097/QAD.0000000000004508

**Published:** 2026-05-28

**Authors:** James Mason, Manik Kohli, Ebun Taiwo, Irfaan Maan, Yomna Gharib, Emmi Suonpera, Maeve Barlow, Flora Olcott, Laura Waters, Alejandro Arenas-Pinto, Richard Gilson, Sarah Pett

**Affiliations:** a Centre for Clinical Research in Infection and Sexual Health, Institute for Global Health, University College London; b Department of Sexual Health and HIV, Central and North West London NHS Foundation Trust, London, England, UK.

**Keywords:** antiretroviral therapy, doravirine, genotypic resistance testing, HIV, HIV resistance, nonnucleoside reverse transcriptase inhibitor, viral suppression

## Abstract

**Background:**

Doravirine is a non-nucleoside reverse transcriptase inhibitor (NNRTI) recommended for first-line antiretroviral therapy (ART), and for suppressed switching, in combination with a two nucleos(t)ide reverse transcriptase inhibitor (NRTI) backbone. Doravirine does not have a high barrier to resistance, and genotypic resistance testing (GRT) preswitch is recommended. Data on its use without preswitching GRT is limited. This retrospective cohort study presents data on doravirine use in virologically suppressed people with and without prior GRT.

**Methods:**

A retrospective single-center review of health records was conducted among prescribed doravirine-based ART at a London HIV service.

**Results:**

Of the 582 adults included, 75% were men, 56% were of White ethnicity. The median age at baseline was 51 years (IQR: 44–58). Median time on doravirine was 127 weeks (IQR: 93–175). GRT showing no doravirine resistance was available for 78% (*n* *=* 454) of individuals before switching to doravirine, of whom 2% experienced virological failure (*n* *=* 9), including two people with NNRTI and NRTI resistance. Among those without preswitching GRT (*n* = 97), 1% experienced virological failure (*n* *=* 1), with NNRTI and NRTI resistance. There was no statistical significant difference in virologic suppression (viral load <50 copies/ml) rates (GRT 95% versus no-GRT 95%; *P* = 0.823). A similar proportion of people with (24%) and without (27%) preswitching GRT switched away from doravirine, most commonly due to adverse effects.

**Conclusion:**

We did not find differences in efficacy and tolerability in people switched to doravirine-based ART with or without GRT. As expected, emergent NNRTI and NRTI resistance was common amongst those with virological failure on doravirine.

## Introduction

Doravirine (DOR) is a non-nucleoside reverse transcriptase inhibitor (NNRTI) recommended in combination with nucleoside reverse transcriptase inhibitors (NRTIs) as a switch and first-line antiretroviral therapy (ART) option for treatment of HIV-1 in current guidelines [1–3]. Characteristic of the NNRTI class, DOR does not exhibit a high barrier to resistance, with as many as 30% and 23% of people experiencing virological failure developing NNRTI and NRTI resistance respectively [4]. As such, RNA or DNA genotypic resistance testing (GRT) prior to DOR initiation is recommended [1–3].

In the absence of historical RNA sequencing, proviral DNA sequencing may inform clinical management. However, these assays might miss some or all previous drug-resistance mutations, and interpretation of results must be done cautiously, as they only reflect proviral archive in peripheral blood mononuclear cells. The CDC recommends proviral DNA genotyping may provide additional information in selected patients with low or undetectable viral loads, although this recommendation is based on limited evidence [3].

There is limited data on the use of DOR-based ART in people with HIV (PWH) where GRT was not undertaken prior to starting DOR. Evidence on real-world use of DOR-based regimens is available [5], but studies have been limited to individuals with documented resistance profiles. This report examined whether virological outcomes following switch to DOR-based ART differed according to the availability of preswitch GRT in routine clinical practice.

## Materials and methods

Data were extracted from electronic health records of all people prescribed DOR for a minimum of 28 days with at least one postswitching plasma HIV viral load between December 2019 and November 2023 at a central London HIV service. Demographics, ART history, all available prior GRT results, and virological and clinical outcomes were recorded. GRTs were defined as those obtained pre-ART on viraemic samples and/or proviral DNA sequencing from peripheral blood mononuclear cells. Resistance tests were conducted using Sanger sequencing. Resistance was classified using the Stanford University HIV Drug Resistance Database [6]. All GRTs were analysed together due to the small number of pro-viral DNA resistance tests (*n* *=* 12) and use of the same clinical interpretation in our real-world cohort.

The overall cohort prescribed DOR was characterized. Only outcomes of patients prescribed DOR-based ART in the context of suppressed switching (viral load <50 copies/ml) were included in the further analysis.

Virological failure was defined as two or more detectable HIV viral loads more than 200 copies/ml or one viral load load more than 200 copies/ml considered as representing clinical failure. Viral “blips” were defined as a single viral load of 50–200 copies/ml preceded and followed by an undetectable viral load (viral load <50 copies/ml) in line with British HIV Association (BHIVA) guidelines [1]. Chi-squared test was used to compare virological outcome data between the patients with and without GRT prior to DOR prescription.

## Results

### Baseline demographics, antiretroviral therapy, and genotypic resistance testing

Five hundred eighty-two people were prescribed DOR-based ART as part of routine HIV care (Table 1). The majority were men (75%), of White ethnicity (56%), with a median age of 51 years (IQR: 44–58). Most individuals were ART-experienced (97%; *n* = 565) with a median number of 3 (IQR: 2–5) previous regimens.

**Table 1:** Baseline demographics and antiretroviral therapy formulation prior and at doravirine initiation.

	Overall (*N* = 582) *n* (%)	Treatment experienced with GRT (*N* = 454) *n* (%)	Treatment experienced without GRT (*N* = 97) *n* (%)
Sex			
Female	142 (24%)	110 (24%)	24 (25%)
Male	439 (75%)	343 (76%)	73 (75%)
Undefined	1 (0%)	1 (0%)	0 (0%)
Ethnicity			
Asian	25 (4%)	18 (4%)	6 (6%)
Black	145 (25%)	121 (27%)	17 (18%)
Mixed	24 (4%)	16 (4%)	8 (8%)
Other	16 (3%)	11 (2%)	5 (5%)
White	324 (56%)	261 (57%)	47 (48%)
Data not available	48 (8%)	27 (6%)	14 (14%)
Age (years)			
18–39	82 (14%)	52 (11%)	21 (22%)
40–49	182 (31%)	144 (32%)	27 (28%)
50–59	207 (36%)	171 (38%)	30 (31%)
60–69	97 (17%)	76 (17%)	17 (18%)
70 and above	14 (2%)	11 (2%)	2 (2%)
ART combination prior to initiating DOR			
Triple therapy			
*2NRTI + NNRTI*	281 (48%)	220 (48%)	61 (63%)
*2NRTI + INSTI*	151 (26%)	125 (28%)	24 (25%)
*2NRTI + PI*	78 (13%)	68 (15%)	9 (9%)
*Other*	7 (1%)	7 (2%)	0 (0%)
Dual therapy			
*PI + NRTI or NNRTI*	12 (2%)	11 (2%)	1 (1%)
*INSTI + NRTI or NNRTI*	18 (3%)	17 (4%)	1 (1%)
Other	7 (1%)	6 (1%)	0 (0%)
Data not available	1 (0%)	0 (0%)	1 (1%)
Naive	17 (3%)	n/a	n/a
Off treatment	10 (2%)	n/a	n/a
DOR formulation at initiation			
DOR/3TC/TDF	498 (86%)	395 (87%)	84 (87%)
DOR/3TC/TDF + INSTI	8 (1%)	3 (1%)	0 (0%)
DOR/3TC/TDF + other	1 (0%)	1 (0%)	0 (0%)
DOR (single agent) in combination with other ART	75 (13%)	55 (12%)	13 (13%)
*DOR + 2NRTI*			
DOR + TAF/FTC	32 (5%)	21 (5%)	10 (10%)
DOR + ABC/3TC	8 (1%)	7 (2%)	1 (1%)
DOR + TDF/FTC	4 (1%)	3 (1%)	1 (1%)
*DOR + other*	31 (5%)	24 (5%)	1 (1%)

3TC, lamivudine; ABC, abacavir; FTC, emtricitabine; INSTI, integrase strand transfer inhibitor; PI, protease inhibitor; TAF, tenofovir alafenamide fumarate; TDF, tenofovir disoproxil fumarate.

Of the 565 ART-experienced patients prescribed DOR, 98% (*n* = 551) were virologically suppressed (<50 copies/ml) prior to switching. Fewer than 1% (*n* = 4) were ART-experienced with a detectable viral load prior to switching to a DOR based ART, and 2% (*n* = 10) were restarting ART after a period off treatment.

Among virologically suppressed people switching to DOR, GRT was available preswitching for 82% (*n* = 454/551), majority of which (*n* *=* 442/454) were RNA and the remaining DNA (*n* *=* 12/454). Median number of previous regimens was 3 (IQR: 2–5) and 51% (*n* = 231/454) were on an NNRTI-based regimen already. No one with GRT prior to DOR initiation had any documented DOR resistance. GRT was not available for 18% (*n* = 97/551), with a median of 3 (IQR: 2–5) previous regimens and 64% already on an NNRTI-based ART (*n* = 62/97). GRT was available prior to DOR prescription for all who were ART-naïve (*n* = 17/17), restarting ART (*n* = 10/10), and ART-experienced with a detectable viral load prior to switching to a DOR based ART (*n* *=* 4/4).

### Virological outcomes on doravirine

Median time on DOR for the cohort (*n* = 582) was 127 weeks (IQR: 93–175). Of these, 86% were prescribed as DOR/lamivudine/tenofovir disoproxil fumarate fixed-dose combination (FDC). Emtricitabine/tenofovir alafenamide fumarate FDC plus DOR was the next most common regimen (5%).

Of those who were virologically suppressed, with preswitching GRT (*n* = 454), 95% (*n* = 433/454) maintained continuous virological suppression (viral load <50 copies/ml), 2% (*n* = 9/454) experienced viral blips or rebounds and re-suppressed without switching ART, 1% (*n* = 3/454) experienced viral blips and switched ART due to other reasons (liver transaminitis; cognitive difficulties; preference for injectable ART), and 2% (*n* = 9/454) experienced virological failure, all of whom had clinician documented poor adherence. Of those who experienced virological failure, all underwent subsequent RNA GRT and two (<1%) demonstrated new resistance. The first had NNRTI (V106 M, E138A, and Y318F) and NRTI (M184 V) resistance, and the second also had NNRTI (K103N, V108I, F227L, and M230L) and NRTI (M184 V and T215 V) resistance.

Among patients without preswitching baseline GRT (*n* = 97) all were virologically suppressed at switching; 95% (*n* = 92/97) maintained continuous virological suppression, 2% (*n* = 2/97) experienced viral blips or rebounds and re-suppressed without regimen change, 2% (*n* = 2/97) experienced viral blips and were switched due to reasons other than virological failure (hospitalization; clinician decision). One person (1%) with clinician documented poor adherence experienced virological failure after 93 weeks; subsequent GRT demonstrated NNRTI (V106A, F227L) and NRTI resistance (V75I, T215C). Of all virologically suppressed individuals, regardless of whether GRT results were available before commencing DOR, none experienced virological failure with good adherence.

**Fig. 1:**
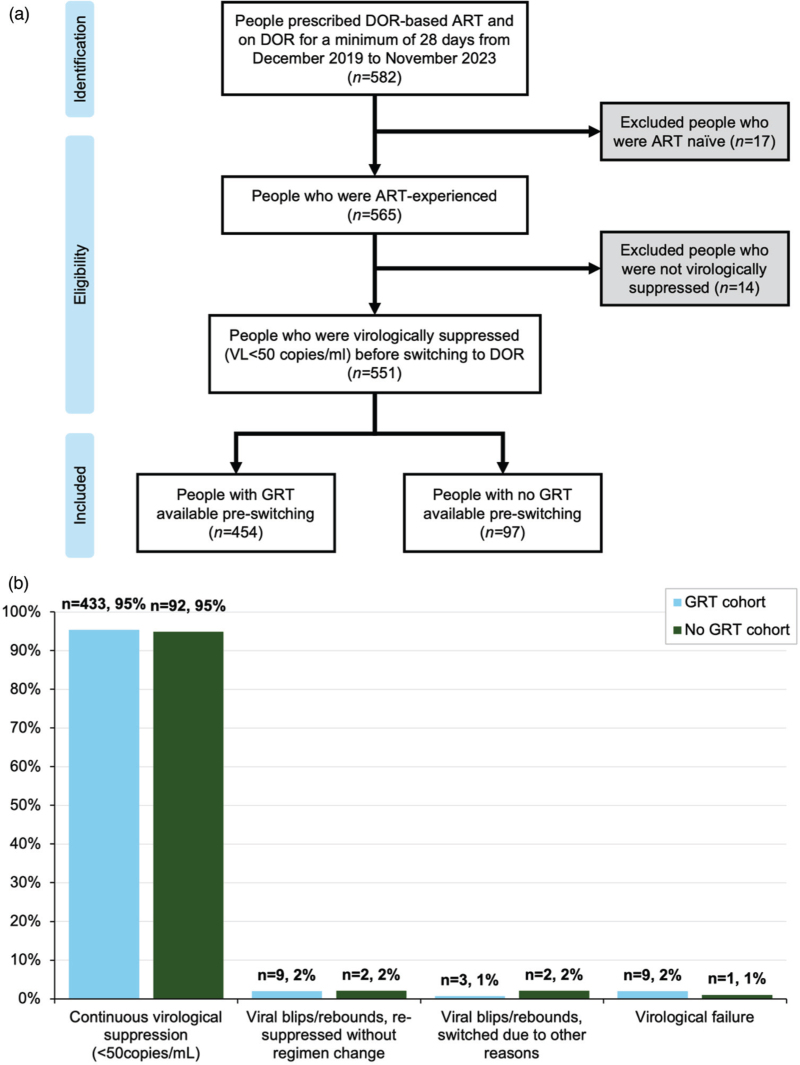
(a) Flowchart of cohort selection. (b) Virological outcomes of virologically suppressed (viral load <50 copies/ml) patients switching to DOR.

Among the people who switched to DOR-based ART with and without baseline GRT (Fig. 1), there was no statistically significant difference in virologic suppression rates (GRT 95% versus no-GRT 95%; chi-squared *P* = 0.823) and no difference in virological failure rates (GRT 2% versus no-GRT 1%; chi-squared *P* = 0.524).

### Reasons for discontinuing doravirine

For virologically suppressed people with preswitching GRT, 24% (*n* = 111/454) switched away from DOR. The majority were due to adverse effects (68%; *n* = 75/111), including mood change (*n* = 14), sleep disturbance (*n* = 13), and gastrointestinal symptoms (*n* = 10). The median time to discontinuation was 50 weeks (IQR: 12–104), with a median of 4 (IQR: 3–6) previous regimen changes.

Of the virologically suppressed people without GRT, 27% (*n* = 26/97) switched away from DOR. The most common reason for switching was adverse effects (69%; *n* = 18/26), including gastrointestinal symptoms (*n* = 5), transaminitis (*n* = 5), and sleep disturbance (*n* = 4). The median time to discontinuation was 51 weeks (IQR: 23–104) with a median of 3 (IQR: 3–6) previous regimen changes.

## Discussion

### Doravirine use in the absence of resistance testing

Current BHIVA guidelines recommend “all newly diagnosed patients should have a baseline resistance test with a genotyping test” [1]. Where historical results are unavailable, proviral DNA resistance testing is advised before switching to regimens with a low genetic barrier to resistance. This reflects the concern that, although DOR retains activity against most common NNRTI resistance mutations, the accumulation of NNRTI-associated mutations may result in VF. In large resistance databases, DOR resistance on GRT was present in almost 85% in whom NNRTIs were not effective [7]. This has led to the recommendation that switching to DOR in patients in whom NNRTIs were not effective should only be considered if GRT shows susceptibility.

Despite these concerns, this study demonstrates DOR appears to maintain high efficacy in the absence of GRT, with a very low incidence of documented VF. Almost 20% of our patients had no GRT prior to switching to DOR, and of these, nearly two-thirds were previously on a NNRTI-based regimen.

In the DRIVE-REAL study looking at real-world utilization of DOR [8], 4% of treatment-experienced individuals had major NNRTI resistance at baseline, of whom all recorded viral load less than 50 copies/ml at 6 months. This is in agreement with posthoc analysis of both MK8591A-017 and MK8591A-018 trials which describe the presence of NNRTI-associated resistance in participants on DOR/islatravir, and in which patients maintained viral suppression regardless of archived mutations [9], and an Italian cohort of ART-experienced individuals switched to DOR-containing regime in the presence of NNRTI mutations [10].

In countries with limited access to viral load monitoring and where resistance tests are seldom available, our findings provide evidence for DOR to be considered as an alternative to dolutegravir in maintaining virological control. However, caution is warranted, especially for PWH with previous NNRTI failure and/or GRT showing reduced sensitivity of NNRTIs who are often excluded from analyses [8,11], and further research of the effectiveness of DOR in the absence of resistance testing is required.

### Doravirine tolerability and side effects

Switching ART regimen in PWH is often necessary to improve tolerability and reduce toxicities [12]. Our study found higher overall DOR discontinuation (27%) compared to other real-world DOR studies (3–8%). Side effects were the leading cause of discontinuation, accounting for approximately 70% of cases. These included sleep disturbance and gastrointestinal symptoms, which are consistent with DOR's side effect profile [13]. However, the patients were highly ART-experienced with a median of 4 (IQR: 3–6) previous regimens. It is possible that reported side effects may reflect cumulative treatment-related intolerability that prompted initial switch to DOR, rather than being solely attributable to DOR itself.

Among those who discontinued, the median time on DOR was 50 weeks for those with a GRT prior to initiation and 51 weeks for those without, indicating discontinuation is largely not immediate. This is consistent with findings from the DRIVE-REAL study, where the median time to discontinuation was 76.5 days.

### Strengths and limitations

The strengths of this analysis are that it uses real-world data from a large cohort of patients on DOR, with long average follow-up times. Limitations include that this study only includes data from a single center in the UK which may limit the generalizability of findings to other clinical settings. In addition, individuals with documented resistance to DOR would not have been switched to DOR-based ART, introducing selection bias in the GRT group. Complete data on prior virological failure on non-DOR NNRTI regimens was not available, which also limits our ability to fully characterize historical NNRTI resistance in the group without baseline GRT. The retrospective real-world nature meant that 6-monthly viral load data was not available for every patient, and heterogeneity in ART history, including NNRTI exposure, may represent confounding factors.

## Conclusion

In our clinical practice, we did not observe differences in efficacy or tolerability when switching to DOR-based ART with or without prior GRT. Virological failure occurred in 1% of virologically suppressed people switching to DOR without baseline GRT compared to 2% of those with baseline GRT. However, consequent emergent NNRTI resistance might limit future ART options.

Despite the guidance regarding preswitch GRTs, in clinical practice, people virologically suppressed on NNRTI-based ART or another regimen with a low barrier to resistance (e.g., raltegravir) are being switched to DOR-based ART. These data demonstrate that this is well tolerated in a selected population where close follow-up is feasible.

DOR discontinuation rates were higher than reported in other real-world cohorts, largely driven by tolerability concerns in a highly ART-experienced population, although discontinuation typically occurred after a prolonged treatment.

Further research could determine whether patients stable on DOR-based ART without prior GRT have archived resistance in the pro-viral DNA and whether this influences the durability of virological suppression.

## Acknowledgements

There was no specific funding for this work.

J.M., M.K., I.M., E.S., R.G., A.A.P., and S.P. conceptualized the study. J.M., E.T., and Y.G. carried out data collection. J.M. analyzed the data. L.W., S.P., A.A.P., M.K. interpreted the findings. M.K., M.B., F.O. prepared the initial draft manuscript. All authors edited and revised the manuscript.

### Conflicts of interest

The authors have no conflicts of interest to declare.
